# Physicians’ understanding of antibiotic intravenous-to-oral switching—a qualitative study in Suzhou, China

**DOI:** 10.1186/s12913-024-11064-2

**Published:** 2024-05-30

**Authors:** Hui Zhang, Junjie Pan, Zhanhong Hu, Jie Pan, Hua Zhang

**Affiliations:** Department of Pharmacy, The Second Affiliated Hospital of Soochow University, Suzhou, China

**Keywords:** Antibiotic, Intravenous infusion, Oral, Qualitative research, Physicians

## Abstract

**Background:**

The implementation of antibiotic intravenous-to-oral switch (IVOS) therapy in hospitals can slow down the development of drug resistance, reduce the occurrence of adverse reactions, and bring significant economic benefits. The aim of this study is to investigate the understanding of physicians at the Second Affiliated Hospital of Soochow University in Suzhou, China towards the antibiotic IVOS therapy.

**Methods:**

15 physicians working in 9 different departments of the Second Affiliated Hospital of Soochow University participated in this study. A semi-structured face-to-face interview was conducted to collect interview information about the antibiotic IVOS therapy. NVivo12 software was used to organize the entire interview content, and the interview data was analyzed and summarized using the Colaizzi seven step method.

**Results:**

60% of participants were not familiar with antibiotic IVOS therapy. Barriers of antibiotic IVOS therapy were included by three key issues: (i) Physicians’ potential cognition: ‘Iv is always better than oral’; (ii) Subjective infusion intention of patients; and (iii) Limitations of drug selection. 60% of participants expressed welcome for pharmacists to help them perform antibiotic IVOS treatment. And electronic recognition technology may be a feasible method for prompting IVOS conversion that recognized by all participants in the interview. Participants also provided some suggestions for pharmacists and IVOS computer reminders.

**Conclusion:**

Physicians’ in China still have insufficient understanding of antibiotic IVOS therapy. The promotion of antibiotic IVOS therapy in China faces many challenges and obstacles. Strategies such as IVOS therapy computer reminders and clinical pharmacists’ medication guidance were worth studying to help physicians develop antibiotic IVOS treatment.

**Supplementary Information:**

The online version contains supplementary material available at 10.1186/s12913-024-11064-2.

## Introduction

Nowadays, antimicrobial resistance (AMR) has become a global threat to the world’s public health system [1]. Hospitals around the world have incorporated antimicrobial stewardship (AMS) into hospital policies to promote the responsible use of antibiotics [2]. Promoting antibiotic IVOS therapy is one of the widely adopted strategies in AMS programs internationally [3]. The term ' IVOS therapy ' is used to describe the early switch of IV to oral therapy once physician determines the clinical stability of the patient according to prescribed standards, without losing antibacterial efficacy [4]. Antibiotic IVOS criteria reported by papers most consist of five-section: (1) timing of IV antibiotic review; (2) clinical signs and symptoms; (3) infection markers; (4) enteral route and (5) infection exclusions [5]. Large-scale implementation of early antibiotic IVOS therapy has been proven to effectively reduce the risk of catheter related infections, increase patient comfort and mobility and save medical costs [6, 7]. Additionally, safety and effectiveness of early antibiotic IVOS therapy compared to the full course use of IV in hospitalized adults have been demonstrated in a large number of clinical studies [8]. IVOS was widely studied in the USA and UK for AMS and even more drug varieties, such as proton pump inhibitor and antineoplastic [9, 10].

The Guiding Principles for the China Clinical Application of Antibiotics clearly give priority to recommend antibiotics with good oral absorption for the majority of patients with mild to moderate infections, except for some patients who are not suitable for oral administration can use IV first [11]. However, the results of the National Medical Services and Quality Safety Report in China show that the proportion of inpatients receiving IV infusion therapy remains high, especially antibacterial drugs, and 70% of IV infusion therapy was unnecessary and can be replaced by other routes of administration [12]. According to statistics by Zhu Bin et al., the total production of large infusion fluids in China in 2019 was 10.5 billion bottles/bag, which is equivalent to using 7.5 bottles/bag of infusion fluids per person per year, far higher than the international average of 2.5–3.3 bottles/bag [13]. A data analysis of 3964 II level and above comprehensive hospitals nationwide in 2020 found that the rate of IV infusion for inpatients in China reached 86.10% [14]. The indicator of “reducing the usage rate of IV infusion in hospitalized patients” has been included in the “China Medical Quality and Safety Improvement Goals” for three consecutive years.

To sum up, the IVOS therapy mode is of great significance for the management of antibiotic IV infusion for inpatients. There has been little study to our knowledge of IVOS relevant research in China. As the holders of prescription rights, physician’s recognition of the switch therapy would directly affect the promotion of the IVOS therapy model, which deserves the attention of researchers and policy. In addition, previous foreign studies have shown pharmacist involvement can effectively optimize the antibiotic use in the process of promoting IVOS policy [15]. Therefore, we performed this study among the physicians in the Second Affiliated Hospital of Soochow University to investigate physicians’ understanding of antibiotic IVOS and identify its driver factors working against early transitions from IV antibiotic to oral, which may be helpful to explore antibiotic IVOS optimization strategy in China.

## Methods

### Setting and research design

The Second Affiliated Hospital of Soochow University is a comprehensive “Grade A, Level III” hospital situated in Suzhou, China, which has 42 clinical departments, 2058 sickbeds and received about more than 2.5 million outpatient and emergency visits and 90 thousand hospitalized cases each year. This study adopts a qualitative and descriptive design to explore physicians’ understanding of antibiotic IVOS therapy in the Second Affiliated Hospital of Soochow University. According to the Consolidated Criteria for Reporting Qualitative Studies checklist, data of this study was collected by semi-structured interview [16].

### Ethical considerations

This study was conducted in accordance with the Helsinki Declaration. The research has been reviewed and approved by the institutional review board of the Second Affiliated Hospital of Soochow University (EC2023245). Informed consent was obtained from all subjects.

### Sampling and recruitment

We sent our interview invitations to clinical physicians of at least 3 years working experience. Individuals who were interested in this study and have well communication skills would been asked to provide their additional information and confirmed the date and time for interview. After giving their informed consent 15 physicians from 9 clinical departments were taken part in a 20–25 min semi-structured face-to-face interview from July 2023 to August 2023. 12 samples were used for data analysis and 3 samples were used for saturation testing.

### Data collection

Interviews were conducted according to an outline, which included four sections: (1) Physicians’ understanding level and pathways of antibiotic IVOS therapy; (2) Barriers of antibiotic IVOS therapy; (3) Physicians’ recognition and advice for pharmacists participating in IVOS therapy; (4) Physicians’ views and advice on implementing IVOS therapy using electronic recognition technology. Data analysis reached saturation on the 12th sample, with the remaining 3 samples used for saturation testing. During the interview, questions were open-ended, and participants were encouraged to freely express their true thoughts. All recordings were recorded through audio recording and transcribed in full and de-identified by two researchers after the interview within 24 h [17]. The translated text was returned back to participants for review to ensure the accuracy and consistency of information.

### Analysis

Subjects in this study were coded as P1–P15. Thematic content analysis was used for the interview recording analysis (Supplementary file1) according to the Colaizzi seven step approach. Researchers used the NVivo 12 software to code the appropriate segments of interview transcripts and classify them into sub-themes and themes based interpretative analysis. This article excerpted some representative statements from the participants.

## Result

A total of 15 physicians were interviewed, including 12 males and 3 females. The average working experience of the respondents was 12 years, coming from 9 different departments. The sociodemographic characteristics of the respondents were shown in Table 1. After analyzing the content of the interviews, four themes about physicians’ understanding of antibiotic intravenous-to-oral switching were extracted as follows:

**Table 1:** Socio-demographic characteristics of study participants

Number	department	professional post	Duration of working
P1	Anesthesia intensive care unit	chief physician	16
P2	Orthopedic surgery	associate chief physician	11
P3	Orthopedic surgery	chief physician	15
P4	Respiration department	chief physician	32
P5	Respiration department	attending physician	9
P6	Urology Department	Resident physician	3
P7	Urology Department	Resident physician	3
P8	Urology Department	associate chief physician	27
P9	Hepatobiliary surgery	associate chief physician	15
P10	Neurology Department	attending physician	3
P11	Neurology Department	Resident physician	3
P12	Nephrology Department	associate chief physician	14
P13	Nephrology Department	attending physician	5
P14	Gastrointestinal surgery	associate chief physician	12
P15	Oncology department	associate chief physician	12

### Theme 1: Physicians’ knowledge of antibiotic IVOS switch therapy

60% of the participants in this study (9/15) mentioned that they were not familiar with antibiotic IVOS therapy. And two people of them stated that they generally only consider switching to oral antibiotic when patients are about to discharged. It is worth noting that 66.7% participants (6/9) this group of physicians were located in the ICU or surgical department. Compared to antibiotic IVOS therapy, they focus more on rapid control of the patient’s condition and surgical treatment.

We are not familiar with antibiotic IVOS therapy and more concerned with surgical treatment in daily work. During the perioperative period of hospitalization, oral medication is generally not considered. We usually only prescribe oral medication to patients upon discharge. (P2)

I’m not familiar with antibiotic IVOS therapy. Many of our physicians’ habits are to give IV fluids to patients during hospitalization for a few days, and then prescribe some oral medication when they are discharged. (P15)

I am not familiar with the treatment of antibiotic IVOS and do not need it. We usually choose IV infusion to control patient’s infection as soon as possible and prevent missing the best treatment opportunity. In general, we do not consider switching therapy during the treatment. If the patient’s infection is well controlled and they no longer have a fever after IV for a period of time, we will directly stop anti-infection treatment. (P11)

40% participants (6/15) stated that they were familiar with the antibiotic IVOS therapy. Among them, both physicians from the respiratory department expressed a strong understanding of antibiotic IVOS therapy and stated that this is a fundamental knowledge that their department’s physicians need to master. Through interviews, we also learned that physicians’ understanding of antibiotic IVOS therapy mainly comes from clinical treatment guidelines and clinical experience.

The criteria for antibiotic IVOS conversion therapy are knowledge that respiratory physicians must master. The relevant knowledge mainly comes from the guidelines. Both HAP and CAP treatment standards have clinical pathways. We strictly follow the recommended indicators in the guidelines to evaluate whether patients can switch from IV to oral, such as c-reactive protein, procalcitonin, white blood cells, imaging features, and clinical symptoms. (P4)

I am familiar with antibiotic IVOS conversion therapy. The downgrading and conversion therapy of antibiotics are conventionally done in my clinical work based on years of treatment experience (P9).

### Theme 2: barriers of antibiotic IVOS therapy

#### Subtheme 2.1‑Potential cognition: ‘IV is always better than oral’

There were 67% participants (10/15) described a recognition in both themselves that IV antibiotics held some kind of mythical status and the efficacy of IV antibiotics was always better than oral. Although some drugs’ instructions indicate that their oral bioavailability is no significant difference compared to IV, physicians are still concerned that switching to oral antibiotics may affect the progression of the disease and lead to worsening of the condition.

In our traditional understanding, the IV antibiotics effect is better and takes effect faster than oral. Especially for some critically ill patients, I may have concerns about switching from IV to oral administration. (P15)

Oral administration needs to pass through the digestive tract and then into the bloodstream. IV administration directly enters the bloodstream and reaches the lesion, so I think the effectiveness of oral administration still needs to be discounted. (P2)

#### Subtheme 2.2‑Subjective infusion intention of patients

There were 47% participants (7/15) described that subjective preferences of patients for use IV influencing their choice of IV versus oral antibiotics. With the progress of society and the improvement of public knowledge, in most cases, patients are willing to accept the suggestion of oral administration if physicians provide it. However, for some elderly patients, it is difficult to reverse their superstition about IV infusion. To avoid complaints, physicians may sometimes choose to respect the patient’s personal willingness to use IV antibiotics.

If the infusion is stopped during hospitalization, some patients may ask, ‘Are you sure I don’t need to continue infusion?’ We usually tell patients that the infusion has been used for a long time, and continuing to use it may result in some adverse reactions, which is not worth the loss. Many patients will agree to stop the infusion according to medical advice. (P5)

I think it’s okay for most patients to switch from IV to oral antibiotics. But some elderly people insist on continuing IV antibiotics, which sometimes makes IVOS difficult. (P10)

From the perspective of patients, especially the elderly, they think IV is necessary during hospitalization. (P7)

#### Subtheme 2.3‑Limitations of drug selection

In the interview, 33% of respondents (5/15) stated that although there were currently many types of antibacterial drugs available, some drugs still did not have oral dosage forms, such as carbapenems and aminoglycosides antibacterial drugs, which limited the switch from IV to oral therapy. In addition, 6.7% of respondents (1/15) stated that medical institutions have formulated corresponding reward and punishment policies for clinical practice in order to achieve the utilization rate of national centralized procurement of drugs. So sometimes physicians may choose more IV antibiotics that are included in the centralized drug procurement list. Above factors influenced the antibiotic IVOS therapy.

There are too many national volume-based procurement drugs now, and some drug formulations are not very complete. Not all drugs can be changed from IV to oral, which is limited by its variety. (P4)

Some drugs do not have oral alternatives. Policy restrictions on drug use also have an impact on IVOS therapy, such as the utilization rate of national volume-based procurement drugs. (P11)

### Theme 3: Physicians’ recognition and advice for pharmacists participating in IVOS therapy

In our hospital, there are about 15 pharmacists in various specialized fields, including anti infection, anticoagulation, nutrition, oncology, and ICU medicine. In this interview, 60% of the respondents (9/15) expressed a great welcome for pharmacists to participate in clinical diagnosis and therapy, believing that they can assist physicians in antibacterial IVOS therapy, provided that pharmacists can provide sufficient evidence-based medical evidence. 33% of respondents (5/15) expressed concern about the effectiveness of the medication recommendations given by clinical pharmacists. They hope that the IVOS treatment recommendations given by pharmacists can ensure effectiveness, with sufficient evidence-based medicine or clinical research results to support it.

We will consider the antibiotic IVOS treatment suggestions proposed by pharmacists. But the treatment plan requires evidence, and providing relevant evidence-based medical evidence is necessary to convince us. (P10)

If the antibiotic IVOS treatment suggestions proposed by pharmacists have been proven to guarantee the treatment effect of patients through practice, we will consider accepting their suggestions. (P11)

13% of respondents (2/15) stated that the timeliness of pharmacist intervention was very important. The changes in clinical conditions are complex, and pharmacists can only provide the most effective advice in the first time by closely following the patient’s diagnosis and therapy process.

Clinical practice places great emphasis on timeliness. If I were to prescribe medication to a patient now but have to wait for pharmacist advice, it may affect our efficiency (P1).

One of the interviewees stated that clinical pharmacists should communicate more with physicians about treatment plans, master pharmacokinetics and pharmacology knowledge proficiently, and improve their professional technical level in order to better provide pharmaceutical services.

To be honest, the professional level of clinical pharmacists needs to be improved. Currently, most pharmacists do not have the ability to guide physicians in medication. The development of clinical pharmacy abroad is quite good, and it is worth learning from. I hope that pharmacists can proficiently master pharmacokinetic knowledge, understand the patient’s condition and clinical knowledge, and provide medication recommendations based on drug instructions and evidence-based evidence. (P4)

### Theme 4: Physicians’ views and advice on implementing IVOS therapy using electronic recognition technology

There were researches reported that foreign hospitals use hospital information systems to mark relevant prescriptions and send intervention measures to physicians, prompting them to make decisions (whether to switch or not). If the physician refuses to switch without reasonable reasons, the pharmacist will resend the IVOS prompt through the system [15]. Currently, the IVOS switching intelligent prompt function has not been introduced in China. This study introduced the respondents to this IVOS management method abroad and consulted their views on implementing this management method in our hospital. All respondents (15/15) believe that implementing IVOS therapy using electronic recognition technology is feasible, and they also put forward some of their own suggestions on that.

53% of respondents (8/15) mentioned that just IVOS electronic prompts are not enough. They also need pop-up information can take into account the complexity of different disease types in different departments, that includes follow-up treatment recommendations, conversion treatment basis and guideline recommendations.

In practical situations, the condition of each department is different. It is hoped that IVOS treatment recommendations can take into account the characteristics of each department and provide us with detailed medication recommendations. (P3)

In clinical practice, the patient’s condition is complex. I suggest that placing the subsequent medication recommendations based on corresponding guidelines or expert consensus information in the pop-up prompt content. (P11)

47% of respondents (7/15) hope to combine IVOS switch therapy electronic recognition with on-site communication with clinical pharmacists, believing it to be more flexible and acceptable. In addition, these interviewees stated that it is necessary for pharmacists to carry out clinical publicity and education on antibiotic IVOS therapy, as raising awareness can lead to behavioral changes.

It is impossible to make conclusions only based on system prompts. I hope pharmacists can share us with knowledge related to antibiotic IVOS therapy and their advice. (P1)

I usually don’t pay much attention to the prompts on the information system, and I hope to combine it with the on-site education of clinical pharmacists. (P8*)*

33% of respondents (5/15) mentioned that they hope after the IVOS intelligent switch prompt, physicians will not be forced to undergo switch therapy. Because the patient’s condition cannot be simply judged based on the various examination indicators collected in the medical record system. Excessive mandatory intervention measures may increase the complexity of clinical diagnosis and reduce the efficiency of physicians’ work.

Clinical patients are ever-changing, and everyone’s situation is different, which cannot be generalized. Therefore, I do not want to be forced to execute IVOS after receiving system prompts. (P4).

There are many assessment indicators for antibiotics now, including DDD and DRG, and sometimes policy formulation is inconsistent with our clinical considerations. I hope that the IVOS switch therapy electronic recognition are not mandatory, and do not increase the clinical burden and complexity of prescribing medical orders (P14).

In addition, 13% of respondents (2/15) suggested that the frequency of electronic message prompts for IVOS switch therapy should not be too high, as this will affect their work. 13% of respondents (2/15) mentioned that if IVOS treatment is included in departmental performance evaluation indicators, the execution effect will be better.

## Discussion

Antibiotic resistance and overuse of intravenous fluids are the prominent medical quality issues in China, and the IVOS therapy model is one of the effective management measures proven by researches for that. Early IVOS in patients eligible for oral administration has been confirmed could optimize the use of antibiotics, reduce catheter related infections and healthcare expenses without affecting clinical outcomes [2, 18, 19]. It can not only reduce medical staff’s workload, but also increased patient mobility and comfort. However, physicians’ understanding and attitude towards the IVOS therapy directly affect the implementation of switch therapy in China. This study conducted semi-structured interviews with 15 physicians from 9 clinical departments of the Second Affiliated Hospital of Soochow University. We found that: (1) 60% of participants (9/15) stated that they are not familiar with antibacterial IVOS therapy; (2) The implementation of IVOS was prevented by three key issues: (i) Physicians’ Potential cognition: ‘Iv is always better than oral’; (ii) Subjective infusion intention of patients; and (iii) Limitations of drug selection. 3) 60% of respondents (9/15) believe that pharmacists can assist physicians in implementing IVOS and provide them with medication advice; The high professional level and timely provision of advice by pharmacists are crucial in this process; 4) All respondents believe that using electronic recognition technology to prompt physicians to implement IVOS treatment is feasible. However, the mandatory and cumbersome nature of IVOS electronic prompts is a concern for them. In addition, the pop-up prompt information would be more helpful if it could be combined with the advice of clinical pharmacists and guidelines to provide targeted follow-up treatment recommendations for different infections.

### Reasons for Physicians’ negative attitude towards IVOS

Although early IVOS has developed relatively mature abroad and was recommended for AMS by National Start Smart in the UK and CDC Core Elements of Hospital Antibiotic Stewardship Programs in the USA, 60% of physicians in this study were not familiar with it and not actively perform IVOS in clinical practice. The Largest barrier to antibiotic IVOS therapy was perceived to be physician’s misunderstanding that that IV anything hold better effect than oral. This is related to physicians’ long-term medication habits and limited knowledge of the safety and effectiveness of IVOS conversion, what makes them less confident in implementing early IVOS treatment for patients, fearing a recurrence of the condition. In addition, whether they place more emphasis on clinical judgment rather than published data, or whether they believe that guideline recommendations are not suitable for their patients is also a potential issue. Because the changes in clinical conditions are complex. Therefore, it is necessary to strengthen education and utilize clinical data and evidence-based evidence to encourage physicians to fully recognize the benefits of IVOS conversion therapy and promote IVOS conversion rates. For example, hospitals should establish a specialized management working group composed of medical, clinical, pharmaceutical, nursing, quality control, information, and other departments to develop IVOS treatment plans or clinical pathways, ensure a timely switch therapy. In addition, pharmacist can organize the absolute bioavailability information of oral antibacterial drugs and develop a list of drugs with comparable effects between oral and IV. In departments with a high usage of IV antibiotics, IVOS pilot work should be carried out to collect the safety and effectiveness of patients with different diseases undergoing IV to oral treatment.

In addition, 47% of participants described that patients’ subjective preferences for IV infusion influenced their treatment process, which was similar to the results of a foreign study on the factors affecting IVOS disorders [20]. Patients may have high expectations for therapy effectiveness when admitted, and often consider whether IV injection is necessary for their condition. Due to the increase in complaint culture and litigation volume, many physicians dare not stand up and refuse patients’ requests for IV antibiotics, especially for elderly patients. Medical chart reminder, education and guideline/protocol has been proven by some studies could significantly improve the IV/PO ratio and increase patients’ awareness of rational medication use [21].

Finally, the unavailability of oral varieties of some antibacterial drugs was the objective reason why physicians were unable to implement IVOS, especially for anti-gram negative bacteria drugs. Therefore, this also suggests that in future research, we should take into account the “accessibility of oral sequential therapy varieties” in the IVOS conversion indicators and not impose restrictions on the autonomy of clinical physicians in prescribing.

### Physicians’ recommendations for antibacterial IVOS therapy management

In this study, 60% of respondents welcomed pharmacists to provide them with IVOS treatment recommendations, based on their recognition of the long-term work of clinical pharmacists. Physicians expressed the hope that pharmacists can regularly participate in clinical case discussions and introduce the latest evidence-based medical evidence and research findings to them, so that they can understand the latest medication knowledge beyond their clinical experience. The professional knowledge level of pharmacists and the ability and timeliness of communication with physicians are very important. Furthermore, all respondents believe that applying electronic recognition technology to IVOS computer reminders can bring positive results. Of note, computer reminders should be paired with experienced pharmacists to provide IVOS treatment advice, otherwise it is difficult to cope with complex clinical situations [22]. And the functional design of computer reminders should be emphasized, including the frequency of reminders and the actions required to reject reminders, otherwise the workload and resistance of physicians may increase. In addition, the medication recommendations for IVOS treatment should also be reflected in concise text in computer prompts to facilitate physicians in determining subsequent medication plans. These details were not mentioned in the previous research on antibiotic IVOS management intervention measures.

### Advantages and limitations

This is the first study in China to investigate physicians’ knowledge and attitude of antibiotic IVOS. The barriers and physicians’ management recommendations on early IVOS in clinical practice were revealed in this study, which providing reference for further improving IVOS management strategy. The research results fill the gap in qualitative research on IVOS in China and have profound implications for regulating IV infusion of antibiotics. This study also provides reference significance for international readers to conduct research on the influencing factors of physician prescription behavior. Due to the differences in healthcare systems, cultural environment and development level among countries, some advanced health management strategies such as antibiotic IVOS cannot be directly copied and used. Qualitative research in the early stage is necessary, which may allow the project more widely applicable and accessible. However, the limited source and number of respondents in this study also bring certain limitations. For example, the research object was only physicians from one hospital, which was limited by the development status of research institutions and regions, and couldn’t effectively represent the opinions of physicians from tertiary hospitals nationwide. In addition, the interview process in this study was conducted in Chinese for communication, but the final output was in English. Although multiple researchers conducted language correction during this period to improve translation accuracy as much as possible, there may still be ambiguity during the language conversion process.

## Conclusion

After the interview, we gained insights into the poor understanding of physicians towards antibiotic IVOS and potential obstacles to that within a level III hospital in China. The IVOS therapy computer reminders and involvement of clinical pharmacists were potential strategies recognized by the majority of participants for promoting antibiotic IVOS therapy, but there were still many details that need to be optimized. Considering of potential obstacles is crucial for developing the IVOS intervention strategies to optimize the use of antibiotics in the future researches.

### Electronic supplementary material

Below is the link to the electronic supplementary material.

Supplementary Material 1

## Data Availability

The dataset generated during and analysed during the current study are available from the corresponding author on reasonable request.

## Abbreviations

IVOS: Intravenous-to-oral switch
AMR: Antimicrobial resistance
AMS: Antimicrobial stewardship
IV: Intravenous
