# Supplementary material for: Physicians’ understanding of antibiotic intravenous-to-oral switching—a qualitative study in Suzhou, China
Source: BMC Health Serv Res. 2024 May 30;24:686. doi: 10.1186/s12913-024-11064-2 (PMC11141074; doi:10.1186/s12913-024-11064-2)
Supplement: Supplementary file 1 — Supplementary Material 1 [file 12913_2024_11064_MOESM1_ESM.docx]

**Physicians' Understanding of Antibiotic Intravenous-to-oral Switching—a Qualitative Study in Suzhou, China**

Hui Zhang^1†^, Junjie Pan^1†^, Zhanhong Hu^1^, Jie Pan^1*^, Hua Zhang^1*^

Affiliations：1. Department of Pharmacy, The Second Affiliated Hospital of Soochow University, Suzhou, China.

Corresponding author: Hua Zhang, [zhsuzhou1977@163.com](mailto:zhsuzhou1977@163.com)；Jie Pan, panzy1122@163.com

**Supplemental table 1:**

**Overview of investigation**

| **Themes** | **Categories** | **Codes** | **Content** |
| --- | --- | --- | --- |
| Physicians'understanding of antimicrobial IVOS therapy | Understanding level | Familiar | *“I am familiar with* *antimicrobial IVOS,I think this is a conventional method. There are not many patients in our department who actually take oral medication because our patients have very serious conditions, and we mainly use intravenous medication. When our patients stabilize their condition and are transferred to a regular ward, we will switch to oral medication.”* (D1) |
|  |  |  | *“The criteria for antimicrobial IVOS conversion therapy are knowledge that respiratory doctors must master. The relevant knowledge mainly comes from the guidelines. Both HAP and CAP treatment standards have clinical pathways. We strictly follow the recommended indicators in the guidelines to evaluate whether patients can switch from IV to oral, such as c-reactive protein, procalcitonin, white blood cells, imaging features, and clinical symptoms.” (D4)* |
|  |  |  | *“I am familiar with* *antimicrobial IVOS, and we also use antibiotic downgrade methods in our daily lives. If the patient's condition improves, intravenous medication will be stopped, or they will take some orally when discharged.” (D5)* |
|  |  |  | *“I am familiar with* *antimicrobial IVOS,* and w*e strictly follow the treatment guidelines for medication use” (D8)* |
|  |  |  | *“I am familiar with antimicrobial IVOS conversion therapy. The downgrading and conversion therapy of antibiotics are conventionally done in my clinical work based on years of treatment experience” (D9)* |
|  |  |  | *“I have a good understanding of the knowledge related to the treatment of IVOS with antibiotics. And I also do this in my daily diagnosis and treatment process. For example, if there is a tumor patient, after a few days of intravenous infusion treatment, I will refer to infection indicators and other factors to convert them to oral treatment or stop taking medication.” (D14)* |
|  |  | Not familiar | *“We are not familiar with antimicrobial IVOS therapy and more concerned with surgical treatment in daily work. During the perioperative period of hospitalization, oral medication is generally not considered. We usually only prescribe oral medication to patients upon discharge.”* (D2) |
|  |  |  | *“I'm not very familiar with it. During hospitalization, we usually don't switch patients to oral medication because intravenous medication is relatively convenient in the ward.”* (D3) |
|  |  |  | *“I'm not very familiar with it.* *Usually, we use medication based on our treatment experience” (D6)* |
|  |  |  | *“I'm not very familiar with it. Usually, we use medication according to the instructions of our superiors and personal experience. And we usually give patients oral medication when they are discharged from the hospital” (D7)* |
|  |  |  | *“I'm not very familiar with it.* *Most of the patients in our department are in a severe coma state, and they are mostly treated with intravenous infusion of antibiotics” (D10)* |
|  |  |  | *“I am not familiar with the treatment of antimicrobial IVOS and do not need it. We usually choose IV infusion to control patient's infection as soon as possible and prevent missing the best treatment opportunity. In general, we not consider switching therapy during the treatment. If the patient's infection is well controlled and they no longer have a fever after IV for a period of time, we will directly stop anti-infection treatment.” (D11)* |
|  |  |  | *“I am not very familiar with this aspect. Our patients are mainly acute pyelonephritis patients, and the treatment of this disease is relatively simple compared to pulmonary infections, so the use of antibiotics ourselves is not particularly professional.” (D12)* |
|  |  |  | *“I don't know much about this area. In fact, our nephrology department is relatively weak in the field of anti infection treatment.” (D13)* |
|  |  |  | *“I'm not familiar with antimicrobial IVOS therapy. Many of our doctors' habits are to give IV fluids to patients during hospitalization for a few days, and then prescribe some oral medication when they are discharged.” (D15)* |
|  | Understanding pathways | Treatment guidelines | *“We strictly follow the recommended indicators in the guidelines to evaluate whether patients can switch from IV to oral, such as c-reactive protein, procalcitonin, white blood cells, imaging features, and clinical symptoms.” (D4)* |
|  |  |  | “w*e strictly follow the treatment guidelines for medication use” (D8)* |
|  |  |  | *“The recommendations for IVOS treatment are explained in the relevant guidelines for anti infection treatment, and I also study them regularly“ (D1)* |
|  |  | Clinical experience | *“The downgrading and conversion therapy of antibiotics are conventionally done in my clinical work based on years of treatment experience” (D9)* |
|  |  |  | *“The knowledge related to IVOS treatment mainly comes from personal clinical experience. I usually decide whether to perform IVOS based on the patient's condition (changes in inflammatory indicators, subjective symptoms, including fever).” (D14)* |
|  |  | Propaganda and education | *“I mainly obtain information about IVOS treatment through propaganda and education” (D5)* |
| Barriers of antimicrobial IVOS therapy | Potential cognition: ‘Iv is always better than oral’ | Bioavailability | *“Oral administration needs to pass through the digestive tract and then into the bloodstream. IV administration directly enters the bloodstream and reaches the lesion, so I think the effectiveness of oral administration still needs to be discounted.” (D2)* |
|  |  |  | *“Our doctors subconsciously believe that intravenous medication is more effective. There are concerns about the effectiveness of switching to oral treatment, which may increase medical risks. Because oral medication needs to pass through the digestive tract and then into the bloodstream, intravenous administration directly passes through the bloodstream to reach the lesion, so I think oral treatment is not as effective as intravenous administration.” (D3)* |
|  |  |  | *“The bioavailability of oral therapeutic drugs is definitely not as high as intravenous infusion. The therapeutic effect may decrease, and the patient's infection may not be well controlled.” (D5)* |
|  |  |  | *“Some of the cephalosporin drugs we commonly use are more effective when administered intravenously. I think intravenous medication takes effect faster and has a higher bioavailability.” (D7)* |
|  |  | Personal habits | *“I personally feel that intravenous medication has a better therapeutic effect” (D6)* |
|  |  |  | *“I have some concerns about the therapeutic effects of some oral medications compared to intravenous infusion. Especially for some patients with severe conditions, doctors may have concerns after switching from intravenous infusion to oral treatment.” (D4)* |
|  |  |  | *“Intravenous antibiotics have a faster onset time, although some oral drug instructions show that they also take effect quickly, I always think intravenous medication would be better.” (D10)* |
|  |  |  | *“In our impression, intravenous medication is often more effective than oral medication.* *Oral medication has a lower bioavailability compared to intravenous medication, and switching to oral medication may also lead to recurrence of the condition.” (D11)* |
|  |  |  | *“In our traditional understanding, intravenous medication is more effective and takes effect faster than oral medication. Based on the condition, if the condition is milder, I may give the patient oral medication. However, complex infections can be more difficult, and administering intravenous medication to patients can take longer.” (D12)* |
|  |  |  | *“In our traditional understanding, the IV antimicrobials effect is better and takes effect faster than oral. Especially for some critically ill patients, I may have concerns about switching from IV to oral administration.” (D15)* |
|  | Subjective infusion intention of patients | Subjective infusion intention of patients | *“From the perspective of patients, especially the elderly, they think IV is necessary during hospitalization.” (D7)* |
|  |  |  | *“Some elderly people may express concerns after switching from intravenous therapy to oral medication. Our doctor will tell him that he doesn't need to continue intravenous infusion in your situation. But sometimes patients may repeatedly request infusion, and doctors are also very helpless.” (D9)* |
|  |  |  | *“I think it's okay for most patients to switch from IV to oral antimicrobials. But some elderly people insist on continuing IV antimicrobials, which sometimes makes IVOS difficult.” (D10)* |
|  |  |  | *“If converted to oral medication during hospitalization, sometimes the patient's family members may question or express a desire to continue infusion.” (D11)* |
|  |  |  | *“Patient factors are also one of the obstacles to antimicrobial IVOS. Some patients may proactively request intravenous infusion” (D13)* |
|  |  |  | *“Patients sometimes have a preference for intravenous infusion, although this has a significant impact on our medication.” (D14)* |
|  |  |  | *If the infusion is stopped during hospitalization, some patients may ask, 'Are you sure I don't need to continue infusion?’ We usually tell patients that the infusion has been used for a long time, and continuing to use it may result in some adverse reactions, which is not worth the loss. Many patients will agree to stop the infusion according to medical advice.” (D5)* |
|  | Limitations of drug selection | Variety restrictions | *“Some antibiotics themselves do not have oral preparations and can only be continuously administered intravenously” (D1)* |
|  |  |  | *“There are too many national volume-based procurement drugs now, and some drug formulations are not very complete. Not all drugs can be changed from IV to oral, which is limited by its variety.” (D4)* |
|  |  |  | *“Some drugs do not have oral alternatives.” (D11)* |
|  |  |  | *“Some drugs do not have oral alternatives in the hospital.” (D12)* |
|  |  |  | *“Some drugs do not have oral alternatives in the hospital, such as sulperazone and piperacillin tazobactam. This will be one of the obstacles to the treatment of antimicrobial IVOS.” (D14)* |
|  |  | Completion rate of centralized drug procurement | *“Policy restrictions on drug use also have an impact on IVOS therapy, such as the utilization rate of national volume-based procurement drugs.” (D11)* |
| Physicians' recognition and advice for pharmacists participating in IVOS therapy | Recognition | Support | *“I definitely strongly support the participation of clinical pharmacists in the diagnosis and treatment process. Because this is helpful for us, we can discuss some new perspectives and ideas with each other.” (D1)* |
|  |  |  | *“I am very willing to accept the medication advice from clinical pharmacists, and I believe it will help improve our work efficiency.” (D2)* |
|  |  |  | *“If clinical pharmacists can provide suggestions for the treatment of IVOS with antibiotics, we would greatly welcome them.” (D3)* |
|  |  |  | *“It would be great if clinical pharmacists could provide some professional medication advice, as they have a better understanding of the pharmacology and pharmacokinetics of drugs.” (D5)* |
|  |  |  | *“We will consider the antimicrobial IVOS treatment suggestions proposed by pharmacists.” (D8)* |
|  |  |  | *“I believe that the participation of clinical pharmacists in the discussion process of medication plans is beneficial for us doctors.” (D9)* |
|  |  |  | *“We warmly welcome clinical pharmacists to provide us with daily medication advice.* *And I hope the pharmacist can introduce us to some of the latest medication guidelines knowledge” (D12)* |
|  |  |  | *“I welcome clinical pharmacists to discuss medication plans with us. I think this is very helpful for us to implement antimicrobial IVOS conversion therapy.” (D13)* |
|  |  |  | *“I am very willing to receive advice from clinical pharmacists on the treatment of antimicrobial IVOS.” (D14)* |
|  |  |  | “It is great to have pharmacists participate in clinical diagnosis and therapy. And we generally recognize the opinions provided by pharmacists.” (D15). |
|  |  | Query | *“To be honest, the professional level of clinical pharmacists needs to be improved. Currently, most pharmacists do not have the ability to guide doctors in medication. The development of clinical pharmacy abroad is quite good, and it is worth learning from. In foreign countries, doctors do not need to think too much about how to use medication, and the medication plan is decided by the pharmacist. However, in China, everything is decided by doctors, and we also hope to have pharmacists involved. However, the professional level and experience of pharmacists are insufficient.” (D4)* |
|  |  |  | *“If the antibiotic IVOS treatment suggestions proposed by pharmacists have been proven to guarantee the treatment effect of patients through practice, we will consider accepting their suggestions.” (D11)* |
|  |  |  | *“If pharmacist proposes an antibacterial IVOS treatment plan and it has been proven to have good therapeutic effects through research, we will consider accepting the relevant medication suggestions from clinical pharmacists.” (D6)* |
|  |  |  | *“It would be a good thing if pharmacists could provide us with some suggestions for the treatment of antimicrobial IVOS, but the prerequisite is to ensure good treatment outcomes.” (D7)* |
|  |  |  | *“We will consider the antimicrobial IVOS treatment suggestions proposed by pharmacists. But the treatment plan requires evidence, and providing relevant evidence-based medical evidence is necessary to convince us.” (D10)* |
|  | Advice | Timeliness | *“And Clinical practice places great emphasis on timeliness. If I were to prescribe medication to a patient now but have to wait for pharmacist advice, it may affect our efficiency.” (D2)* |
|  |  |  | *“Clinical practice places great emphasis on timeliness. If I were to prescribe medication to a patient now but have to wait for pharmacist advice, it may affect our efficiency” (D1).* |
|  |  | Professionalism | *“To be honest, the professional level of clinical pharmacists needs to be improved. Currently, most pharmacists do not have the ability to guide doctors in medication. The development of clinical pharmacy abroad is quite good, and it is worth learning from. I hope that pharmacists can proficiently master pharmacokinetic knowledge, understand the patient's condition and clinical knowledge, and provide medication recommendations based on drug instructions and evidence-based evidence.” (D4)* |
| Physicians' views and advice on implementing IVOS therapy using electronic recognition technology. | Views | Feasible | *“I think it is a good idea.” (D1)* |
|  |  |  | *“I think it is feasible.” (D2)* |
|  |  |  | *“I think it is possible.” (D3)* |
|  |  |  | *“I think it is possible.” (D4)* |
|  |  |  | *“I think this is a good method.” (D5)* |
|  |  |  | *“I think this method is feasible.” (D6)* |
|  |  |  | *“I think this method is helpful in improving our rational use of medication.” (D7)* |
|  |  |  | *“I think this method is acceptable.” (D8)* |
|  |  |  | *“I think this method is quite good.” (D9)* |
|  |  |  | *“I think this method is feasible.” (D10)* |
|  |  |  | *“I think this method is feasible.” (D11)* |
|  |  |  | *“I think this method is helpful.” (D12)* |
|  |  |  | *“I think this method is very good.” (D13)* |
|  |  |  | *“I think this method is feasible.” (D14)* |
|  |  |  | *“I think this method is very good.” (D15)* |
|  | Advice | Detailed | *“In practical situations, the condition of each department is different. It is hoped that IVOS treatment recommendations can take into account the characteristics of each department and provide us with detailed medication recommendations.” (D3)* |
|  |  |  | *“If evidence-based medicine supports the effectiveness of IVOS treatment, doctors would be willing to try it.” (D2)* |
|  |  |  | “It would be even better if the pop-up content provide the conversion treatment basis. For example, we will refer to information such as how long the patient has been taking intravenous medication, and how their blood routine indicators are currently. We will switch to oral medication orders based on this information.” *(D9)* |
|  |  |  | *The current treatment plans are based on guidelines, especially for cerebrovascular diseases, such as how much blood pressure has decreased and whether patients have benefited, which require evidence to support. If the pop-up message content can strictly refer to the guidelines or expert consensus, I believe doctors will be very convinced.” (D10)* |
|  |  |  | *“In clinical practice, the patient's condition is complex. Suggest placing the corresponding guidelines or expert consensus information in the pop-up prompt content.” (D11)* |
|  |  |  | *“Suggested pop-up messages can provide targeted treatment recommendations based on different infection situations, such as multiple infections.” (D12)* |
|  |  |  | *“I hope that the pop-up prompt information can take into account the complexity of different disease types in different departments and provide follow-up treatment recommendations.” (D13)* |
|  |  |  | *“In addition, if the pop-up information could provide follow-up medication recommendations, it would be helpful for us.” (D15)* |
|  |  | Pharmacist involvement | *“It is impossible to make conclusions only based on system prompts. I hope pharmacists can share us with knowledge related to antimicrobial IVOS therapy and their advice.”* (D1) |
|  |  |  | *“However, it is recommended to conduct joint education. Our ward has weekly business learning meetings, and clinical pharmacists can come to promote evidence-based medicine to every physician, so that they can receive IVOS electronic prompts. Because if there is no pharmacist communicating with us in person and only prompts the doctor through a system pop-up, it is highly likely that the doctor will not read the pop-up content and will directly close it. But if the doctor has heard about the relevant educational content, when the pop-up window appears, the doctor may look at the specific content.”* (D2) |
|  |  |  | *“I usually don't pay much attention to the prompts on the information system, and I hope to combine it with the on-site education of clinical pharmacists.”* (D8) |
|  |  |  | *“We hope that clinical pharmacists can go to each department for education and guide us in the use of antibiotics based on pop-up information. When the IVOS treatment concept is deeply rooted in the hearts of every doctor, there may be no need for pop-up messages. In addition, if IVOS treatment is used as a departmental assessment indicator, the effect will be better.”* (D3) |
|  |  |  | *“In addition, the predictive model of IVOS electronic information prompts needs to be scientifically and in detail, providing targeted treatment and medication recommendations for different infection complications, pathogens, patient gastrointestinal function, albumin and other indicators. This requires prediction tools to be validated in a large number of samples. Otherwise, blind and broad pop-up prompts will have no effect.” (D4)* |
|  |  |  | *“In addition, it is recommended that clinical pharmacists go to the clinical department for education and introduce us to the relevant knowledge of IVOS treatment.” (D11)* |
|  |  |  | *“However, pop-up prompts are not flexible enough. It would be better if clinical pharmacists could participate in the discussion of treatment plans on site.” (D15)* |
|  |  | Non mandatory | *“Clinical patients are ever-changing, and everyone's situation is different, which cannot be generalized. Therefore, I do not want to be forced to execute IVOS after receiving system prompts.”* (D4). |
|  |  |  | *“There are many assessment indicators for antimicrobials now, including DDD and DRG, and sometimes policy formulation is inconsistent with our clinical considerations. I hope that the IVOS switch therapy electronic recognition are not mandatory, and do not increase the clinical burden and complexity of prescribing medical orders”* (D14). |
|  |  |  | *“But after the pop-up message prompt, the doctor cannot be forced to change the doctor's order, because the course of treatment with antibiotics for each disease is different.”* (D5) |
|  |  |  | *“In addition, I hope that after the pop-up prompt, I will not be forced to change the doctor's order, nor will I be asked to fill in the reasons for refusing to change the doctor's order in the system, as these will affect our work efficiency.” (D11)* |
|  |  |  | *“In addition, do not force me to change the treatment plan after the pop-up prompt, and do not keep popping up repeatedly, as it will affect my work efficiency.” (D13)* |
|  |  | Do not repeat prompts | *“In addition, I am concerned that pop-up windows may repeatedly appear after being manually closed and cause my electronic medical record system to lag.”* (D5) |
|  |  |  | *“In addition, do not force me to change the treatment plan after the pop-up prompt, and do not keep popping up repeatedly, as it will affect my work efficiency.” (D13)* |
|  |  | Assessment indicators | *“Otherwise, it is difficult for doctors to make changes to their medication habits. In addition, if the treatment of antimicrobial IVOS can be made into indicators, it will be more effective to have the department head push this matter from top to bottom, such as the use rate of centralized drugs, and mandatory implementation will be more effective.” (D2)* |
|  |  |  | *“In addition, if IVOS treatment is used as a departmental assessment indicator, the effect will be better.” (D3)* |
